# Anosmia impairs homing orientation but not foraging behaviour in free-ranging shearwaters

**DOI:** 10.1038/s41598-017-09738-5

**Published:** 2017-08-29

**Authors:** O. Padget, G. Dell’Ariccia, A. Gagliardo, J. González-Solís, T. Guilford

**Affiliations:** 1 Oxford Navigation Group, Department of Zoology, University of Oxford, Oxford, OX1 3PS Oxfordshire United Kingdom; 2 Biodiversity Research Institute (IRBio) & Department of Animal Biology, University of Barcelona, Barcelona, Spain; 3 Department of Biology, University of Pisa, Pisa, Italy

## Abstract

Shearwaters deprived of their olfactory sense before being displaced to distant sites have impaired homing ability but it is unknown what the role of olfaction is when birds navigate freely without their sense of smell. Furthermore, treatments used to induce anosmia and to disrupt magneto-reception in displacement experiments might influence non-specific factors not directly related to navigation and, as a consequence, the results of displacement experiments can have multiple interpretations. To address this, we GPS-tracked the free-ranging foraging trips of incubating Scopoli’s shearwaters within the Mediterranean Sea. As in previous experiments, shearwaters were either made anosmic with 4% zinc sulphate solution, magnetically impaired by attachment of a strong neodymium magnet or were controls. We found that birds from all three treatments embarked on foraging trips, had indistinguishable at-sea schedules of behaviour and returned to the colony having gained mass. However, we found that in the pelagic return stage of their foraging trips, anosmic birds were not oriented towards the colony though coastal navigation was unaffected. These results support the case for zinc sulphate having a specific effect on the navigational ability of shearwaters and thus the view that seabirds consult an olfactory map to guide them across seascapes.

## Introduction

Procellariiform seabirds are some of nature’s greatest navigators. The results of two recent displacement experiments have supported the case that, like pigeons over land^1, 2^, Procellariiform seabirds (albatrosses, shearwaters and petrels) might make use of olfactory information to navigate over large distances across seascapes. In the first displacement^3^, Atlantic Cory’s shearwaters, *Calonectris borealis*, breeding in the Azores were released 800 km in the mid Atlantic ocean and either deprived of their olfactory sense by washing of the olfactory mucosa with zinc sulphate or subject to magnetic perturbation by carrying strong neodymium mobile magnets attached to their heads. Whilst control and magnetically manipulated birds homed successfully along straight routes, birds treated with zinc sulphate wandered in the ocean for thousands of kilometres and were impaired at homing. In a second study^4^, Scopoli’s shearwaters, *Calonectris diomedia*, breeding in the Tuscan archipelago were subject to the same standard treatments and displaced in open sea within the Mediterranean Sea’s basin. In this case, anosmic shearwaters were able to home, but did so significantly more slowly than controls and magnetically manipulated birds. The authors argue that anosmic birds were able to compensate for a lack of olfactory information by making use of the richer topographic information available in the Mediterranean Sea, an idea which was supported by anosmic birds spending significantly more time within 40 km of the coast during homing.

However, while previous studies have shown that magnetic manipulation has no effect on seabird orientation during free-ranging^5, 6^ or homing movement after displacement^3, 4^ it remains unknown how anosmia might affect birds’ orientation during natural, free-ranging movement when given a choice to embark on their own foraging trips or how magnetic or anosmic treatments might affect birds’ ability to forage and gain body mass. The reliance of navigation on olfactory cues has never been tested in a natural context and as such the applicability of discoveries from displacement experiments to natural movement is an open question^7^. A further motivation for examining the effect of navigational treatments (especially anosmia) on free-ranging birds is that disorientation experiments have a limited ability to separate the effect that treatments might have on non-specific factors not directly related to the navigational system that might be the cause of poorer homing performance^8^. Experiments testing the effect of zinc sulphate treatment on information processing in homing pigeons^9, 10^ have not found evidence of a generalised effect, and similarly in seabirds, Dell’Ariccia and Bonadonna^11^ showed that anosmic Cory’s shearwaters retain motivation to home from short-distance displacements, but return during daylight instead of during the night, suggesting a reliance on visual cues in the absence of olfactory information constituting an impairment in navigational ability but not of other non-specific responses to zinc sulphate treatment. However, it remains to be elucidated whether there is a more general behavioural effect of the zinc sulphate treatment in free-ranging and foraging pelagic Procellariiform seabirds and over long-distance orientation.

In this study we aimed at addressing these questions by investigating whether standard navigational treatments used in displacement studies, zinc sulphate induced anosmia^3, 4, 11–13^ and magnetic disruption using strong rare-earth magnets^4, 5, 14^, cause disruption of natural foraging trips in a shearwater. Unlike previous studies, treatments were not followed by translocation. Instead, we tracked shearwaters’ free-ranging foraging trips using miniature GPS loggers. Whilst the current experimental design does not allow us to disentangle interactions among treatments that might preclude inferences on the sensory basis for navigation itself, it did allow us to observe whether manipulated birds were motivated to embark on foraging trips, able to forage effectively and could perform normal behavioural tasks. Furthermore, it allowed us to investigate the effect of olfactory deprivation, achieved by the same means as in previous release experiments, on orientation in free-ranging foraging trips for the first time. Our magnetic manipulation was only partially successful because most birds had lost their magnets by the time they returned from foraging, limiting the robustness of inferences about the impact of magnetic disruption on foraging or the role of magnetic cues in orientation.

## Materials and Methods

### Experimental treatments

Scopoli’s shearwaters’ nest attendance was monitored from 25^th^ June 2016 until 21^st^ July 2016 (during the last third of incubation) at the colony of Cala Morell, Menorca, Spain (40°03′19.2″ N 3°52′55.6″ E) where there is an on-going study of this species, and incubating pairs suitable for manipulation and device deployment were identified. At this stage, new birds were ringed and morphometric measurements taken. Birds were caught either by hand or with a neck noose and were returned to the entrance to their nest crevice and observed after handling. Birds were assigned to treatments alternately. In our first treatment, shearwaters were made anosmic (n = 10) by washing of the olfactory mucosa via the nares (the nostrils on the bill of a bird) with 4 ml of 4% zinc sulphate heptahydrate dissolved in water as in previous studies^3, 4^. Zinc sulphate treatment acts by causing necrosis of the nerve cells in the olfactory mucosa and birds remain anosmic until the cells of the mucosa have differentiated, matured and connected to the olfactory bulb, a process that takes many weeks in the homing pigeon^15^. In our second treatment, birds were disrupted magnetically (n = 10) by attachment of a small cylindrical (4 mm × 5 mm) neodymium magnet secured at the end of a 2 cm × 0.4 cm, flattened cylinder of TESA tape (total mass 1 g) and attached to feathers on the top of the head, directly between the eyes. The magnetic end of the TESA cylinder was free to move around its arc to either side of the bird’s head. Preliminary observations of the anosmic treatment were made on four birds at their nests to assess whether the zinc sulphate treatment negatively impacted incubation behaviour (three of these later entered the experiment, alternated with controls and magnetically disrupted birds). As in Gagliardo *et al*.^3^ control birds (n = 12) were not sham treated with respect to the zinc sulphate treatment since there is robust evidence that washing the olfactory mucosa with saline solution^4^, physiological solution^11, 16, 17^, or the non-olfactory nasal mucosa with zinc sulphate^18^ does not affect the behaviour of petrels but nonetheless the procedure does inevitably increase the risk of inducing some damage to the olfactory mucosa^18^ and could produce a partially anosmic control treatment. However, a glass bead was deployed as a sham in place of a magnet on all non-magnetically manipulated birds so that we could compare controls with both other treatment groups and reduce the number of birds used for the experiment.

Before being treated, birds were fitted with Mobile Action I-gotU gt-120 GPS devices housed in waterproof heatshrink plastic. Devices were attached using TESA tape which was laid underneath small bunches of contour feathers centrally on the back of the birds and wrapped over the device. Housed GPS devices (including TESA) weighed 18 g (3.1% of body mass at the time of deployment, 3.3% including the magnetic and sham treatments) and measured approximately 9 cm × 3 cm × 1.5 cm, which includes tabs of heatshrink housing at each end used for attachment^19^. Birds were weighed immediately prior to the treatment and upon retrieval. GPS devices were scheduled to take fixes every 5 minutes. Total handling time was normally less than 15 minutes. All experimental procedures were conducted in accordance with animal welfare regional legislation (BOIB 97 Decret 65/2004) and were approved by Oxford University’s local ethical review process. Experiments were carried out under licence from the Balearic government (CEP 22/2016).

### Defining the start of homing, at-sea behaviour and foraging success

GPS tracks regularly comprised multiple trips made by the birds between deployment and retrieval. These were split and each trip analysed separately. We employed a multi-step process to identify objectively the homing sections of each trip. Tracks were interpolated by cubic splines such that locations were at exactly 5 minute intervals^20^ before being divided into behaviourally consistent units by implementation of a Douglas-Peucker line segmentation algorithm as in Thiebault and Tremblay^21^. By going backward through each trip, we could then identify the first behaviourally consistent segment that resulted in significant homeward movement. We defined the start of that segment as the decision to home. All pre-processing was carried out blind with respect to treatment.

To identify at-sea behaviour, we fitted a Gaussian mixture model to speed and turning angles calculated for the tracks as in Fayet *et al*.^22^. We identified the optimal number of behavioural states by assessing the log-likelihood of 1–10 states before assigning each GPS location to its most likely state in the best mixture model. Mass gained at sea was determined from the mass change of birds between device deployment and retrieval. Because following deployment birds often continued incubating for several days before departing on a foraging trip, during which time they will have lost mass, we corrected the measured mass change to account for the lower mass of the bird at departure. To do this, we calculated the rate of percentage mass loss for a subset of incubating birds which were weighed more than once during an incubation stint (n = 8) and used this to estimate a departure mass for each of our birds, thus giving a more accurate estimate for the mass gained at sea. We report this as corrected mass gained. We then compared the proportion of GPS locations assigned to each behaviour out of the total GPS locations for day-time and night-time sections of track. Night-time was identified as the time between the end of one nautical twilight and the beginning of the next (calculated for the median GPS latitude and longitude on the median date).

### Outbound and homing orientation

Outbound orientation was the virtual vanishing bearing^23^ of each bird’s position as it reached 10 km on the outward stage of trips to the Catalonian coast, measured between geographical north, the colony and the bird. Homing orientation was analysed separately for the portion of trips that were either coastal (within 40 km of the Balearic archipelago) or pelagic (beyond 40 km of the Balearic archipelago). 40 km was chosen in line with previously published analyses^4^ as the point at which birds could probably see land. For pelagic homing, inbound orientation was defined as being from the identified start of homing to the point where the bird reached the 40 km threshold. Coastal homing was the remainder of the trips from the moment they passed within 40 km of the Balearic coast until they reached the colony (or the entire trip for birds which did not travel further than 40 km from the coast during the entire trip). For both sections, the bearing with respect to home between consecutive interpolated GPS locations identified as ‘flight’ was compared among treatments. Track straightness was measured as the path length between the start of homing and the point where the bird reached 40 km from the Balearic coast divided by the beeline distance (the shortest Great Circle distance between the start and end of the track section).

### Statistics

To test for an effect of treatment on outbound orientation, we conducted a circular analysis of variance (Watson-Williams test). To deal with repeated measures caused by multiple trips from each bird, we iteratively sampled one measure from each bird at random and then performed a Watson-Williams test on this subset. This procedure was repeated 5000 times. We present the estimated mean, standard error (s.e.) and estimated p-values of these iterated Watson-Williams tests.

We used linear mixed models (LMMs) to test for an effect of treatment on homing orientation, the relationship between homing departure time and distance to the colony at the start of homing, total distance travelled in each trip, trip duration, the straightness of return tracks and trip repeatability characteristics (see supplementary material). For LMMs, treatment was coded as a three-level factor (anosmic, magnetically manipulated and control). We used binomial generalised linear mixed models (GLMMs) to test for an effect of treatment on the proportion of time spent in each behavioural state, both for daytime and night-time activity^22^. Each of the three behavioural states was analysed with a separate GLMM, with the response variable being 0 or 1 for each GPS location with treatment, coded as a three-level factor, as a predictor. LMMs and GLMMs included a random intercept effect to account for repeated measures. This was bird ID for response variables calculated on an entire trip (total trip distance, trip duration, straightness of return track), and was trip ID nested within bird ID for response variables measured at each GPS location (orientation home, behavioural state of each GPS location) to reflect the structure of our data. For the GLMMs testing the effect of treatment on the proportion of fixes in each behavioural state, a second random intercept effect was included to account for date effects such as moon state and weather variation across the tracking period. This was the Julian date (for models testing day fixes) or the Julian date at the start of the night (for models testing night fixes) when data were recorded.

To obtain p-values from mixed models, we conducted a likelihood ratio (LR) test between each full model (with treatment and random intercepts effects) and a nested, null case of the model (random intercept effects only). For parameter estimates, models were fitted with restricted maximum likelihood but for LR tests they were refitted by maximum likelihood estimation^24^. Where significant, LMMs were followed by a *post-hoc* Tukey test to evaluate between which levels of treatment significant differences lay with degrees of freedom adjusted by the Satterthwaite method^25^. For LMMs, the assumption of approximately normal residuals was checked by examination of each model’s Q-Q plot. GLMMs were checked for over-dispersion by comparing the sum of squared Pearson residuals to the residual degrees of freedom.

A General Linear Model was used to analyse the relationship between mass gained (between device deployment and retrieval) and time spent foraging, which was estimated from the number of GPS fixes identified as foraging (1 GPS location = 5 minutes). To assess whether treatment affected the mass gained per unit time we ran a GLM with treatment (coded as a three-level factor), time spent foraging and the interaction between treatment and time spent foraging as predictors.

All statistics were conducted using R base or the lme4 package^26^ in R (ver. 3.2.1). Tukey tests were conducted using the multcomp package and the Watson-Williams test was conducted using the circular package.

### Data availability

The data used in this study are available on  Movebank (www.movebank.org, study name “Free-ranging anosmic, magnetic and control Scopoli’s shearwaters”) and are published in the Movebank Data Repository with doi: 10.5441/001/1.c741t5b6.

## Results

### Tracking success and impact of tracking on breeding success

**Figure 1:**
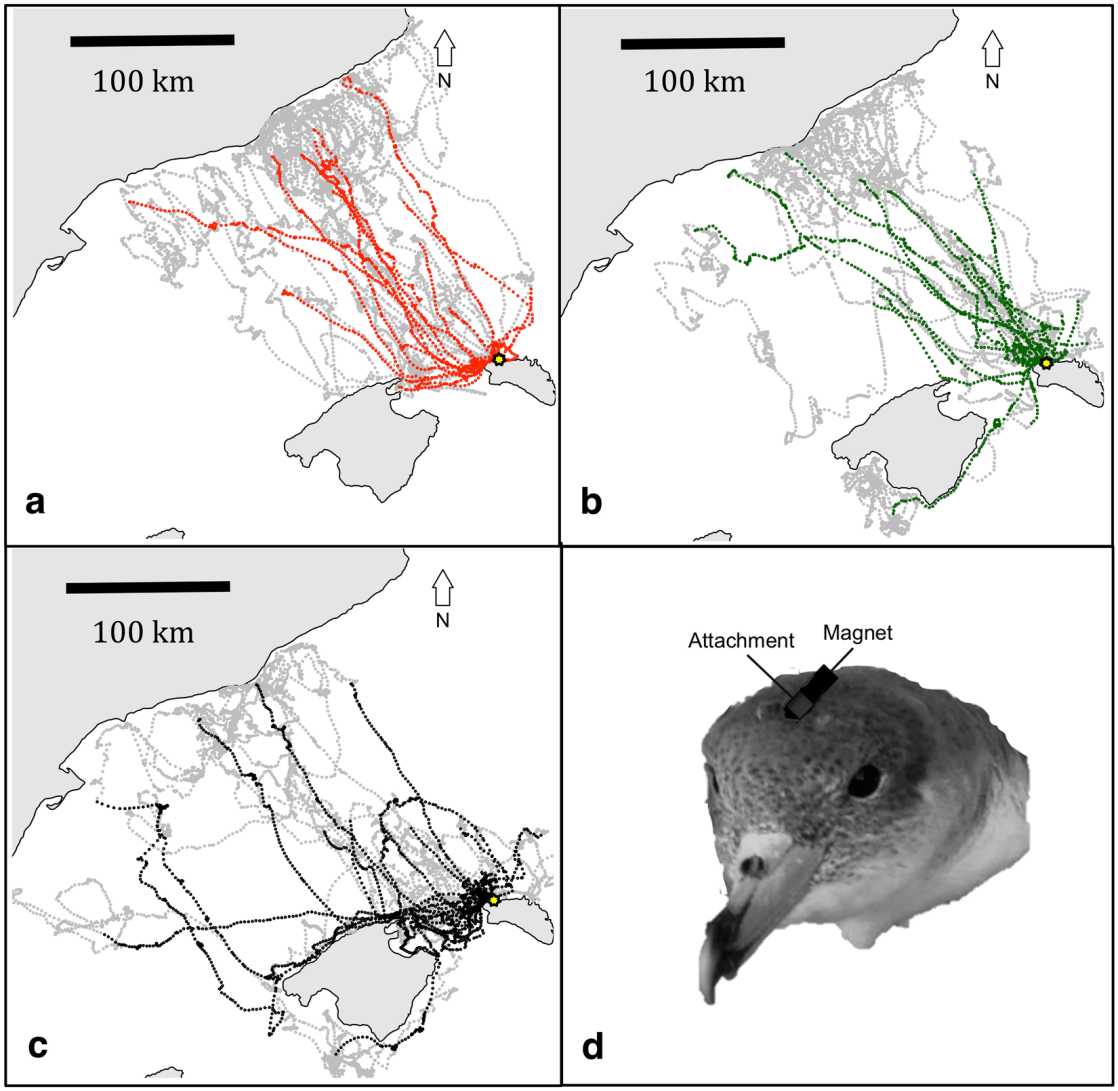
GPS tracks (59 trips) of control (**a**), magnetically manipulated (**b**) and anosmic (**c**) birds. The homing portion of the trips are highlighted for control (red), magnetically disrupted (green) and anosmic (black) birds respectively. (**d**) Schematic showing the TESA magnet attachment to the head of the shearwater consisting of a flattened cylinder of TESA tape attached at the base to the feathers on the top of the head and containing a magnet free to move at the end.Yellow points show the colony position. Maps were generated in R using the “maps” package (Original S code by Richard A. Becker, Allan R. Wilks. R version by Ray Brownrigg. Enhancements by Thomas P Minka and Alex Deckmyn. (2016). maps: Draw Geographical Maps. R package version 3.1.1. http://CRAN.R-project.org/package=maps) and “maptools” package (Roger Bivand and Nicholas Lewin-Koh (2016). maptools: Tools for Reading and Handling Spatial Objects. R package version 0.8–39. http://CRAN.R-project.org/package=maptools).

Deployments for all three treatments were carried out between the 29^th^ June and 10^th^ July and retrievals were made between the 6^th^ and 20^th^ July. 12 control (C), 10 magnetically manipulated (M) and 9 anosmic (A) birds were tracked resulting in tracks from 10 control, 9 magnetically manipulated and 9 anosmic birds comprising a total of 59 foraging trips (16 control, 23 magnetically manipulated and 20 anosmic; Fig. 1). A single GPS carried by a magnetically manipulated bird failed to record data for the full duration of the deployment due to its battery discharging. The resulting incomplete trip was not included in the analysis. All birds that were handled, including those made anosmic, resumed incubation immediately following the procedure and continued to incubate normally until embarking on a foraging trip. All birds embarked on foraging trips and all birds except one magnetically treated bird were seen again within the period that the colony was monitored for returns (until 21^st^ July). Anosmic birds were recovered and GPS devices were retrieved within 15 days of treatment and deployment, a duration over which zinc sulphate-treated birds are assumed to have remained entirely anosmic. Therefore, all trips recorded by the GPS were included in our analyses.

There was no difference in the date of deployment (Kruskal-Wallis test:*χ* ^*2*^ = 0.35, df = 2, p = 0.84), the date of the first outbound trip (Kruskal-Wallis test: *χ* ^*2*^ = 2.15, df = 2, p = 0.34) or the time spent on the nest between deployment and departure (Kruskal-Wallis test: *χ* ^*2*^ = 2.10, df = 2, p = 0.35) among treatments. There was also no difference in the number of trips recorded per individual among treatments (Kruskal-Wallis test: *χ* ^*2*^ = 2.24, df = 2, p = 0.33). Primarily, incubation stint changeovers occurred normally, but birds in all treatments were sometimes recorded leaving the nest 1–3 days before partners returned (1 control, 3 magnetically manipulated and 2 anosmic), a behaviour occasionally observed in crevice-nesting petrels^27^. Of the head-mounted manipulations, only 4/12 control birds and 3/9 anosmic birds and 4/10 magnetically manipulated birds were carrying their sham- or true magnet upon retrieval. After conducting exploratory statistics to test for differences between magnetically treated birds that did and did not retain their magnets (Table S1, supplementary material), magnetically treated birds were pooled together for all analyses.

All nests included in the study were checked for breeding status on 24^th^ July, after hatching had begun. Of the 32 nests included in the experiment, 14 failed to hatch their egg (control 4/12; magnetic 6/10; anosmic 4/10), primarily because of egg predation from brown rat, *Rattus norvegicus*. This did not differ significantly among treatments (Kruskal-Wallis test: *χ* ^*2*^ = 2.15, df = 2, p = 0.34).

### Trip chracteristics, at-sea behaviour and foraging success

#### Trip characteristics

**Figure 2:**
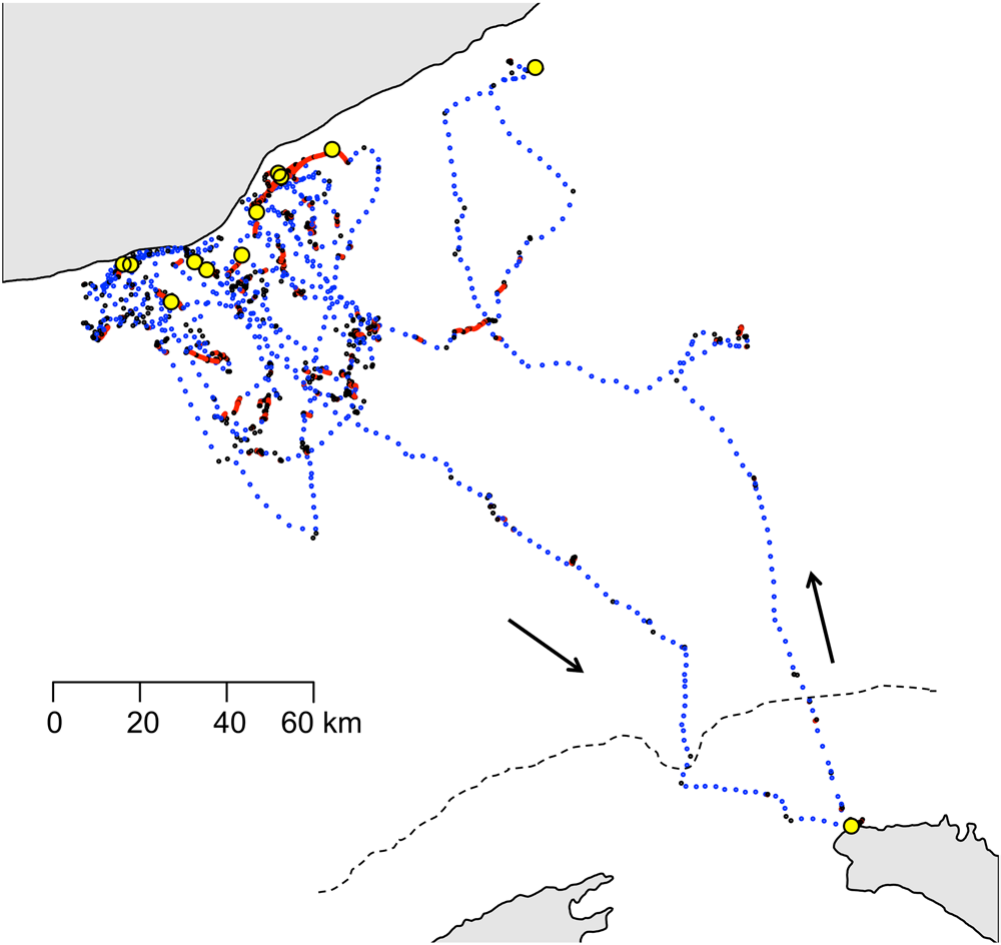
A typical foraging trip to the Balearic coast. Behaviours identified by our mixture model are commuting (blue), foraging (black) and resting (red). Yellow dots show where the bird was at midnight on each day of the trip including the night it departed. This magnetically manipulated bird spent 10 days at sea before commuting back to Menorca. The dashed line shows the 40 km contour from the Balearic Islands. Maps were generated in R using the “maps” package (Original S code by Richard A. Becker, Allan R. Wilks. R version by Ray Brownrigg. Enhancements by Thomas P Minka and Alex Deckmyn. (2016). maps: Draw Geographical Maps. R package version 3.1.1. http://CRAN.R-project.org/package=maps) and “maptools” package (Roger Bivand and Nicholas Lewin-Koh (2016). maptools: Tools for Reading and Handling Spatial Objects. R package version 0.8–39. http://CRAN.R-project.org/package=maptools).

The Scopoli’s shearwaters in this study made long (3–10 day) foraging trips, typical of incubating *Calonectris* shearwaters^28^. The trips mostly comprised an outbound and inbound commute, separated by intense foraging in a relatively restricted area where they most likely foraged on small pelagic fish and cephalopods^29^ caught at or near the surface in plunge dives^30, 31^. A typical, annotated trip is shown in Fig. 2. Foraging trips in this study were broadly made to three areas: the Catalonian coast (n = 20), coastal destinations within 40 km of the Balearic Islands (n = 11) or non-coastal destinations (>40 km offshore) in the Balearic Sea (n = 27). Birds which commuted to the Catalonian coast foraged approximately 35 ± 0.2 km (mean ± s.e.) from the coast between the Ebro Delta (40°42′29.3″ N 0°52′8.0″ E) in the south and the Muntanyes de Begur National Park in the north (Lat: 41°58′46.6″ N 3°16′58.5″ E), a region known to be a foraging hotspot for Mediterranean Procellariiformes^32^. A single anosmic bird travelled to the coast near Valencia (40°4′1.2″ N 0°19′0.8″ E). There was no difference in the propensity of the three treatments to undergo trips to the different regions (*χ* ^*2*^ = 4.11, df = 2, p = 0.128), the duration of foraging trips (LMM estimates ± s.e.: control: 126 ± 37 h; magnetic: 94 ± 27 h; anosmic: 102 ± 27 h; LR test: *χ* ^*2*^ = 0.95, df = 2, p = 0.62), or the distance covered during foraging trips (LMM estimates ± s.e.: anosmic (9 birds, 20 trips) = 867 ± 197 km; control (10 birds, 16 trips) = 1,074 ± 271 km; magnetic (9 birds, 23 trips) = 716 ± 267 km; LR test: *χ* ^*2*^ = 2.11, df = 2, p = 0.35). There was no difference among treatments in the route fidelity between the outbound and inbound sections of birds’ trips (Table S2, supplementary material) or in individual repeatability characteristics of outbound route and destination for birds that underwent multiple trips during the experimental period (Figure S2 and Table S3, Supplementary Material).

#### At-sea behaviour

**Figure 3:**
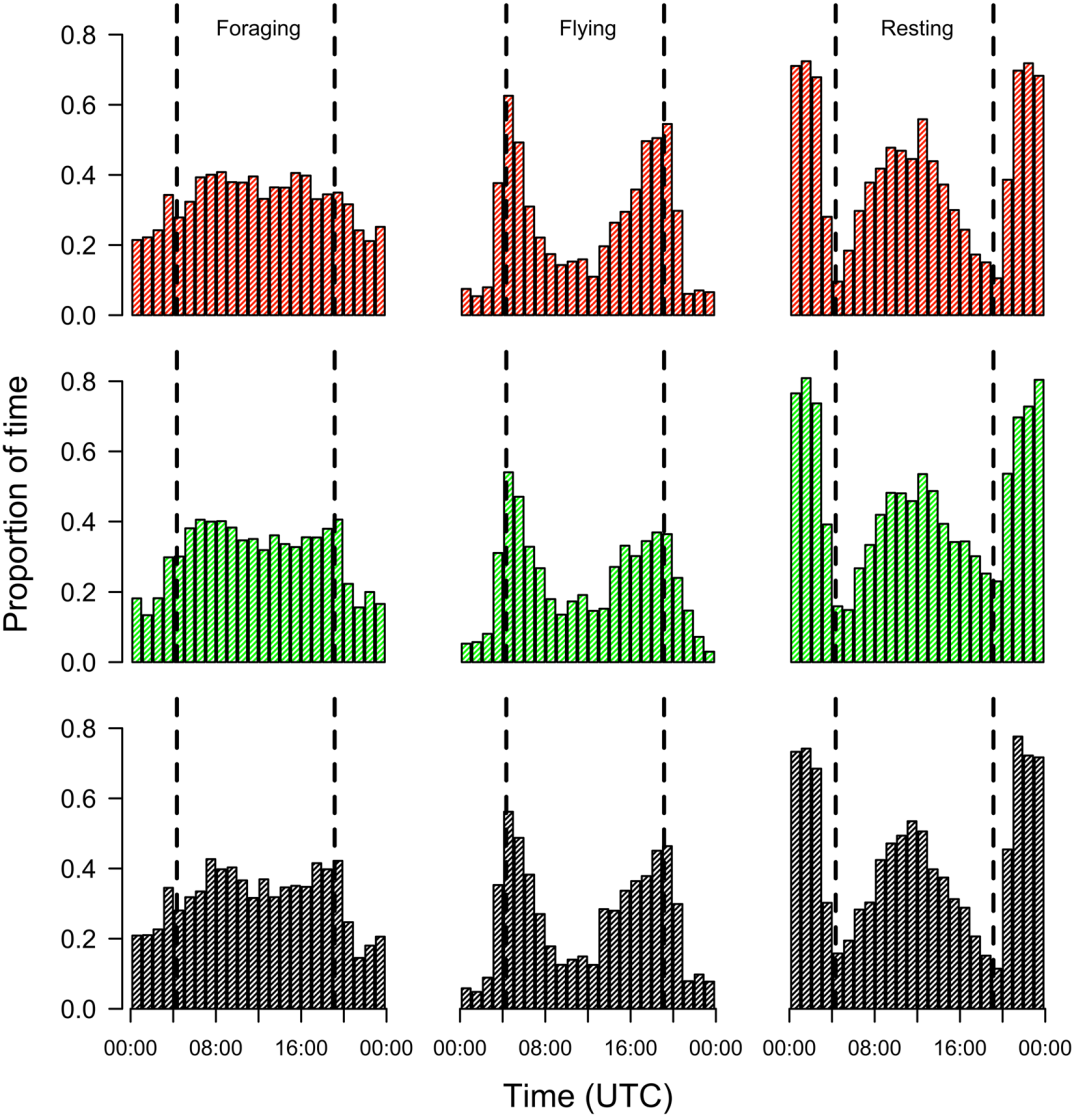
Diurnal behaviour patterns for control (red), magnetically manipulated (green) and anosmic (black) birds. Columns, from left to right are foraging, flying and resting on the water. Sunrise and sunset are shown as dotted lines (times shown are in UTC).

Our mixture modelling approach identified three behavioural states (Figure S1). Since log-likelihood increases monotonically with the number of states assumed, we chose the number of states indicated by an elbow in the increasing log-likelihood as providing the best explanatory power without over-fitting states to reflect real clustering in the data. We interpreted these three states as resting on the water (mean speed = 0.3 ms^−1^), directed flight (mean speed = 6.7 ms^−1^) and foraging (mean speed = 1.8 ms^−1^). There was no significant difference in the proportion of time spent in any of the identified behavioural states among treatments either for behaviour during the day or during the night (Table 1). The diurnal pattern of behaviours was similar across all three treatments (Fig. 3), with the majority of commuting behaviour occurring around dawn and dusk, and more resting on the water at night.

**Table 1:** The proportion of time spent in each behavioural state for the three treatments during day and night.

	Diurnal activity	GLMM		Nocturnal activity	GLMM	
	Mean ± SE	*χ* ^*2*^	p	Mean±SE	*χ* ^*2*^	p
Foraging	Control: 0.37 ± 0.15	0.26	0.88	Control: 0.27 ± 0.14	4.30	0.12
	Magnetic: 0.35 ± 0.16			Magnetic: 0.20 ± 0.13		
	Anosmic: 0.36 ± 0.17			Anosmic: 0.22 ± 0.14		
Resting	Control: 0.31 ± 0.15	2.89	0.24	Control: 0.60 ± 0.14	1.00	0.61
	Magnetic: 0.36 ± 0.16			Magnetic: 0.67 ± 0.13		
	Anosmic: 0.33 ± 0.17			Anosmic: 0.66 ± 0.15		
Commuting	Control: 0.32 ± 0.15	2.86	0.24	Control: 0.13 ± 0.10	0.30	0.86
	Magnetic: 0.29 ± 0.15			Magnetic: 0.13 ± 0.11		
	Anosmic: 0.31 ± 0.16			Anosmic: 0.11 ± 0.11		

Proportions shown are the second order means for the treatments ±s.e. *χ* ^*2*^ and p-values from LR tests are shown.

#### Foraging success

**Figure 4:**
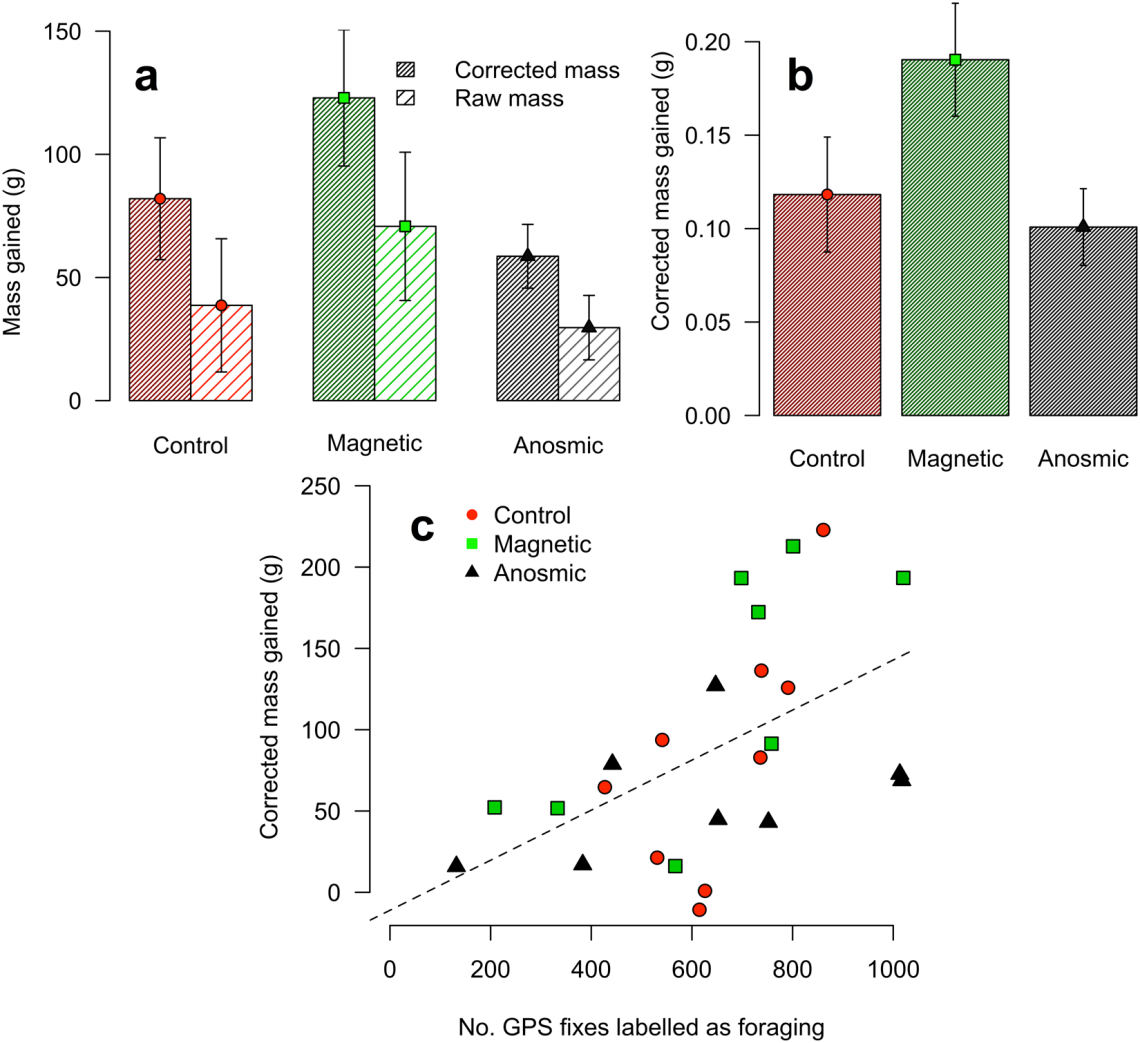
(**a**) Raw mass (mean ± s.e.) change over the course of the deployment and corrected mass (mean ± s.e.) taking account mass lost during incubation between weighing and the start of the trip. (**b**) The corrected mass (mean ± s.e.) gained per GPS location labelled foraging and (**c**) the mass gained as related to the amount of time spent foraging (number of GPS locations labelled foraging). For statistics see text.

Birds from each treatment for which we had pre- and post-deployment mass gained mass on average during the experiment (controls (n = 9): 38.7 ± 27.0 g; magnetic (n = 8): 70.8 ± 30.1 g; anosmic (n = 8): 29.7 ± 13.0 g). There was no significant difference among treatments in the mass gained during the experiment (ANOVA: F = 0.73, df = 2,22, p = 0.49, Fig. 4a). Corrected mass gained (taking account of mass lost while incubating before departure) was also not significantly different among treatments (mean ± s.e.: control (n = 9): 82.0 ± 24.7 g; magnetic (n = 8):  = 122.9 ± 27.7 g; anosmic (n = 8): 58.6 ± 12.9 g; ANOVA: F = 1.94, df = 2,22, p = 0.17; Fig. 4a). Corrected mass gained was significantly predicted by the number of GPS locations labelled as being in the foraging state (GLM: F = 8.57, df = 1,23, p = 0.01; Fig. 4c) but neither treatment nor the interaction between treatment and foraging effort improved the model significantly (treatment: F = 2.63, df = 2,22, p = 0.10; interaction: F = 2.78, df = 2,22, p = 0.09) meaning that there was no effect of treatment on the rate of mass gained per unit foraging effort (Fig. 4b and c).

### Outbound and homing movement

#### Outbound orientation

**Figure 5:**
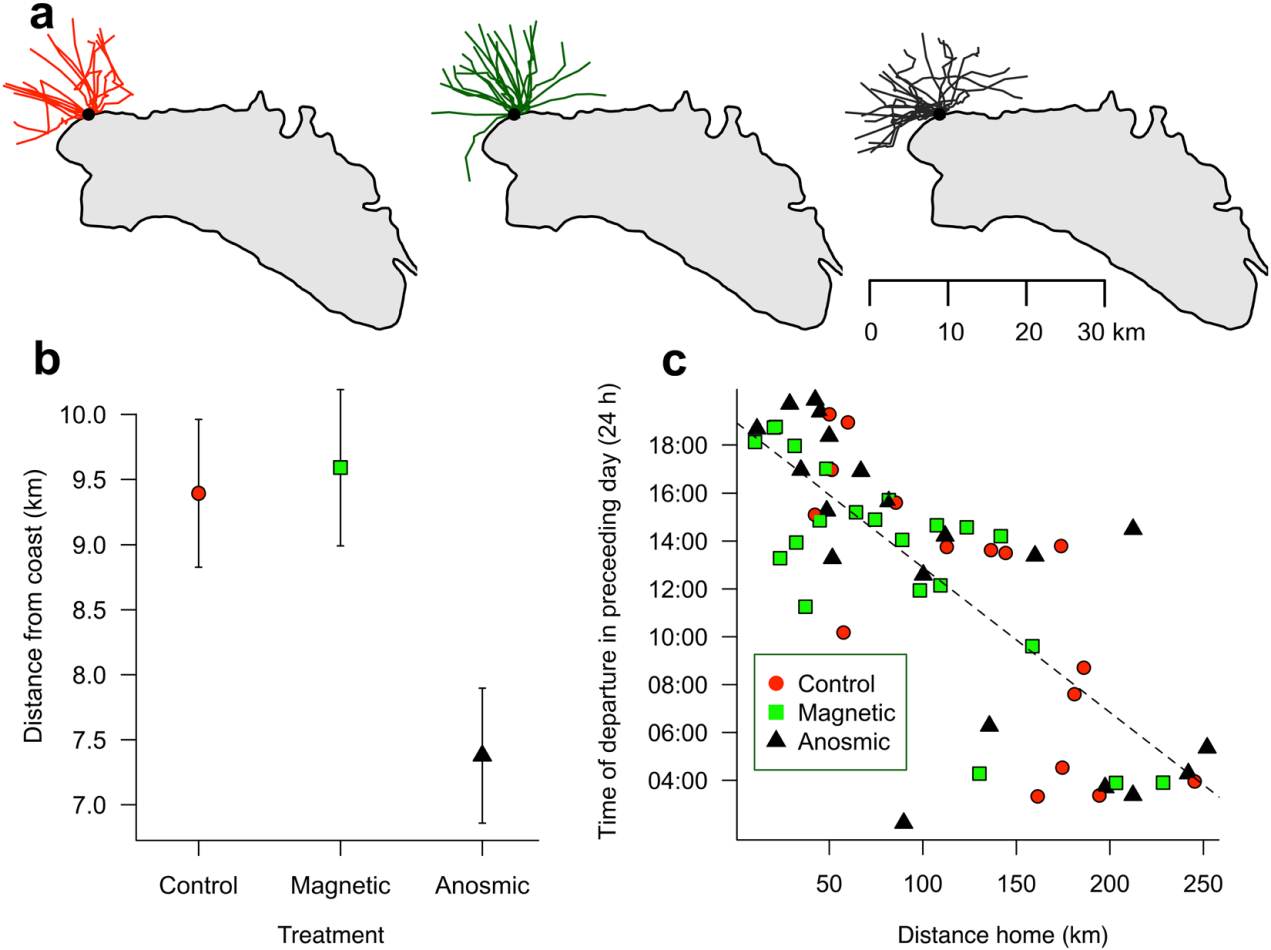
(**a**) Outbound sections of birds’ trips up to 10 km from the colony are shown for anosmic (black), magnetic (green) and control (red) treated birds. (**b**) Distance from Balearic coast as birds reach 10 km from the colony on the outbound sections of trips. (**c**) Time that birds begin to the home to the colony in the day preceding the night of arrival as a function of distance to the colony at the start of homing. Linear regression is shown (see text for LMM statistics). Maps were generated in R using the “maps” package (Original S code by Richard A. Becker, Allan R. Wilks. R version by Ray Brownrigg. Enhancements by Thomas P Minka and Alex Deckmyn. (2016). maps: Draw Geographical Maps. R package version 3.1.1. http://CRAN.R-project.org/package=maps) and “maptools” package (Roger Bivand and Nicholas Lewin-Koh (2016). maptools: Tools for Reading and Handling Spatial Objects. R package version 0.8–39. http://CRAN.R-project.org/package=maptools).

Outbound orientation was not significantly different among treatments (mean directions: control = 316.97°; magnetic = 321.32°; anosmic = 285.23°; 5000 runs of Watson-Williams Test, p = 0.25 ± 0.002, Fig. 5a). However, anosmic birds were initially more coastal in the outbound sections of their trips, being on average ~2 km closer to the coast than magnetically disrupted or control birds as they reached 10 km from the colony (LMM estimates ± s.e.: control: 9,395 ± 843 m; magnetic: 9,637 ± 761 m; anosmic: 7,378 ± 557 m; LR test: *χ* ^*2*^ = 9.73, df = 2, p = 0.01; *post-hoc* Tukey pairwise test: control-magnetic: t = 0.30, p = 0.95; control-anosmic: t = 2.416, p = 0.04; anosmic-magnetic: t = 2.97, p = 0.01; Fig. 5b).

#### Timing of homing

The time that birds began homing on the day preceding the night of arrival (Fig. 5c) was strongly predicted by the distance that they had to travel to reach the colony (LMM LR test: *χ* ^*2*^ = 56.40, df = 1, p < 0.0001) but neither treatment nor the interaction between treatment and distance significantly improved the model (treatment LR test: *χ* ^*2*^ = 1.03, df = 2, p = 0.60; interaction LR test: *χ* ^*2*^ = 0.38, df = 2, p = 0.83), meaning that the ability to anticipate the distance home did not differ among the three treatments.

#### Pelagic orientation home

**Figure 6:**
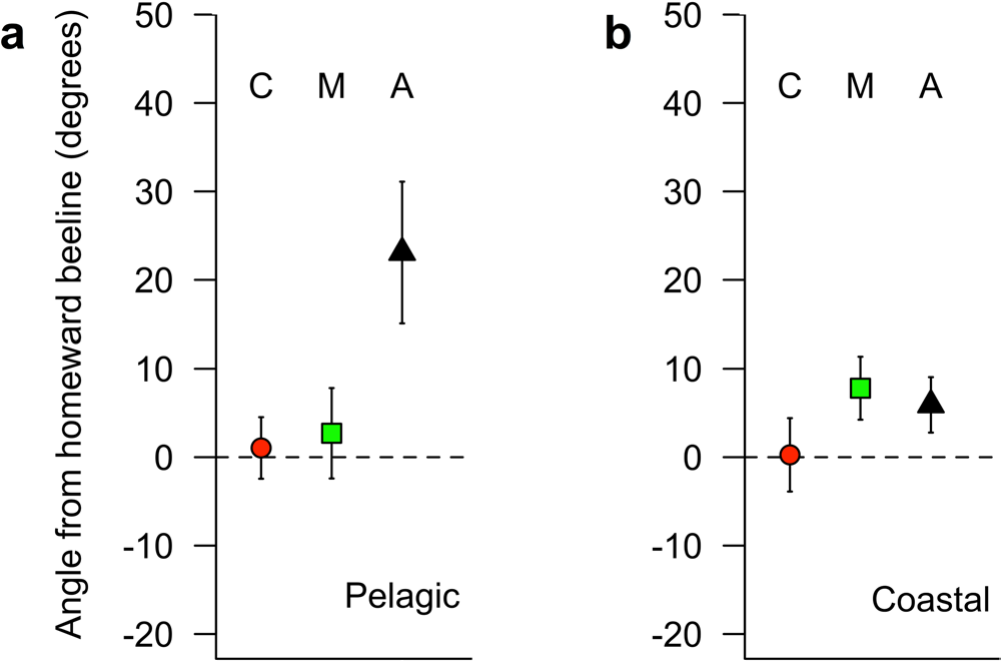
Orientation (mean ± s.e.) during the homing phase (transformed so that homeward direction = 0). Pelagic sections (**a**) are those from the point of homing until 40 km from the Balearic Archipelago. Coastal (**b**) is homing within 40 km of the Balearic archipelago. For statistics see text.

For the pelagic (>40 km from the Balearic coast) sections of trips identified as homing, orientation relative to the beeline home, measured for each GPS location during homing, differed significantly among treatments (LMM estimates ± s.e.: control (n = 12) = 4.3 ± 6.8°; magnetic (n = 14) = 1.7 ± 6.7°; anosmic (n = 12) = 21.9 ± 5.0°; LR test *χ* ^*2*^ = 10.00, df = 2, p = 0.0067; Fig. 6a). A *post-hoc* Tukey test revealed that this difference lay between anosmic and control (t = 2.97, p = 0.040) and anosmic and magnetic (t = 3.02, p = 0.013) but not control and magnetic (t = 0.41, p = 0.912) treatments. For both control and magnetically manipulated birds, the home direction, 0°, lay within the 95% confidence interval for the return orientation whereas for anosmic birds, 0° lay outside of the 95% confidence indicating that during the pelagic homing phase anosmic shearwaters were not oriented toward the colony.

#### Coastal orientation home

Within 40 km of the Balearic archipelago there was no difference among treatments in homeward orientation (LMM estimates ± s.e.: control = 8.9 ± 5.3°; magnetic = 1.4 ± 5.0°; anosmic = 7.0 ± 3.5°; LR test: *χ* ^*2*^ = 2.23, df = 2, p = 0.3282; Fig. 6b). For this coastal phase, the home direction lay within the 95% confidence intervals for all treatments.

#### Track straightness

There was no significant difference in the straightness of the homing trajectory among treatments during the pelagic phase of homing (control = 0.89 ± 0.04; magnetic = 0.93 ± 0.04; anosmic = 0.87 ± 0.03; LR test: *χ* ^*2*^ = 2.22, df = 2, p = 0.33).

## Discussion

In this study, we investigated for the first time the effect of induced anosmia on the free-ranging foraging trips of a pelagic seabird and the effect of magnetic disruption by attachment of strong rare-earth magnets on birds’ ability to forage and gain mass at sea. Both anosmic and magnetically treated shearwaters were able to embark on foraging trips, forage successfully to gain mass, and return to the colony to resume incubation. Given that only four of our magnetically manipulated birds retained their magnets for the entire trip, we cannot draw robust inferences on the effect of the magnetic treatment on the at-sea behaviour of shearwaters in this study. However, it is likely that magnets were retained for the outbound sections of the track which occurred within the first 6 hours of birds leaving the colony and so we confine our inference to effects of the treatment on the outbound sections of birds’ trips.

By inferring at-sea behaviour from clustering patterns in speed and tortuosity data, we were able to compare at-sea behaviour among our treatments and show that both anosmic and magnetically manipulated birds were similar to controls in their ability to perform general behavioural tasks. Gaussian mixture models and other similar pattern recognition techniques have previously been employed to identify at-sea behaviour in other shearwater species and their effectiveness at identifying foraging behaviour has previously been validated using concurrently deployed dive-loggers^33, 34^. In the present study, we confirmed our expectation that birds would gain significantly more mass at sea with more time identified as foraging, implying that our mid-speed behaviour was indeed related to foraging behaviour. In this way we were able to measure mass gained per unit of time foraging at sea and compare this among treatments, allowing us to observe that anosmic and magnetically manipulated birds could gain mass at sea and, importantly, that they did not need to spend more time foraging to do so. Whilst magnetically manipulated birds did gain more mass on their foraging trips than control and anosmic birds, this difference was not greater than that expected by chance and, to our knowledge, there are no plausible ways in which magnetic disruption could increase a bird’s foraging ability.

The foraging success and similarity of nycthemeral patterns in at-sea behaviour between anosmic birds and the other two treatments suggests that the zinc sulphate treatment did not measurably affect birds’ ability to perform normal behavioural tasks. Our choice not to sham-treat control birds with respect to the zinc sulphate treatment does not impact the inferential power of this result, since it is therefore known that control birds did not have reduced foraging ability that could be attributed to the physical procedure of nasal irrigation. The ability of birds treated with zinc sulphate to perform generalised behavioural tasks is an important consideration when interpreting the results of anosmic release experiments^35^. The lack of a generalised response to zinc sulphate at sea expands upon the finding that Cory’s shearwaters retain motivation for short-distance homing and resume normal incubation after treatment with zinc sulphate^11^ and is consistent with previous experiments in homing pigeons. When treated with zinc sulphate solution, pigeons retain their ability to perform forced-choice memory tasks^10^, and are able to home when unilaterally (one nare) irrigated with zinc sulphate providing that they are able to detect atmospheric odours through the second nare^9^. Our finding that anosmic shearwaters are able to time the initiation of their homing trips appropriately according to the distance that they had to travel further suggests that general cognitive processes underpinning navigation remain intact during zinc sulphate treatment. Moreover, that anosmic birds did not differ from controls in their track straightness indicates that anosmic birds’ ability to maintain a heading, most probably by reference to a compass^36^, also remained intact.

While we did not find any generalised effects of our sensory manipulations, we did find some specific effects of zinc sulphate treatment on pelagic orientation. The homing trajectories of anosmic birds during the pelagic phase of homing differed significantly to control and magnetically treated birds with anosmic birds’ orientation not including the homeward direction, 0°, in their 95% confidence interval as they homed to within 40 km from the Balearic coastline. This was a strong effect, present in pairwise *post-hoc* tests with a conservative p-value correction for false positives. The effect of zinc sulphate treatment on orientation was restricted to the homing stage as we found no difference among treatments in the direction of the initial outbound stage of birds’ foraging trips. However, anosmic birds were significantly more coastal (7.4 km for anosmic, 9.4 km for control and 9.6 km for magnetically disrupted birds) upon reaching 10 km from the colony as they departed on foraging trips, implying that shearwaters might make use of visual information more in the absence of olfactory cues. Our magnetic treatment had no effect on outbound orientation or distance to the coast relative to controls, during which time we expect that birds were still carrying their magnets; a finding that is consistent with studies reporting no effect of strong rare-earth magnets on free-ranging orientation in albatrosses^5, 6^ or homing following displacement in white-chinned petrel or Cory’s shearwaters^3, 4, 14, 37^. It is possible that the navigational effect of our anosmic treatment could be caused by the physical discomfort caused by the treatment procedure, since control birds were not nasally irrigated at all. However, the normal behaviour of anosmic birds leading up to the onset of homing extends findings from previous studies which show that there is no impact of nasal irrigation with inert solutions on procellariiform behaviour^9, 11, 16, 17^, constituting good evidence that our effect on homing orientation was caused by a lack of olfactory information rather than non-specific effects of the treatment which could include demotivation to home, poor foraging ability or an inability to time daily activities.

The finding that anosmic birds were motivated to embark on normal, successful foraging trips but nonetheless had impaired orientation during directed homing flight supports the view that birds make use of olfactory information during navigation back to their colony after foraging and is consistent with a navigational interpretation of previous displacement experiments in *Calonectris* shearwaters^3, 4^ and other experiments across a broad range of avian taxa^1, 13, 38^. However, this is the first time that experimental evidence for a role of olfactory information in navigation has been found in a free-ranging bird and thus represents a significant advance in our understanding of pelagic navigation. In this study, we were able to see that olfactory information was not crucial to free-ranging movement within what is likely to be birds’ familiar areas but that an olfactory map is nonetheless consulted for the return leg of these trips even when birds are auto-displaced. Furthermore, that anosmic birds’ orientation recovered as birds came within 40 km of the coast and that anosmic birds remain closer to land during outbound sections of foraging trips suggests that in the absence of olfactory information, shearwaters make use of visual topographic landscape features to guide their movement, a finding which is consistent with previous release experiments in which anosmic shearwaters spent significantly more time during homing within 40 km of the coast than controls^4^ and the shift from night to day homing from short-distance displacement in order to find the nest while anosmic^11^. A peculiar result in this study, therefore, is that birds homing from the Catalonian coast were sufficiently well oriented initially to reach the Balearic archipelago but had poorer orientation than controls despite birds regularly embarking on their homeward trips from the Catalonian coast, an area rich with topographic information. This could be indicative of relatively poor spatial resolution in a bird’s visual map further away from the colony, an assertion further supported by the strikingly parallel orientation in five of the six anosmic birds’ tracks whilst homing from the Spanish coast, possibly indicative of birds simply heading away from the coast after foraging. Alternatively, birds might not make use of visual landmarks at all at the beginning of their homeward journey to the colony perhaps resorting to a compass bearing associated with the end of foraging or tied to knowledge of the outbound journey. However, if this were the case, one might expect some within-trip fidelity between the outbound and inbound sections of trips; an expectation not observed in the current study (see supplementary material).

Like other Procellariiformes^39, 40^, Scopoli’s shearwaters are known to be attracted to dimethyl sulphide at sea and probably locate pelagic resources by olfaction^40^. Given that anosmic shearwaters in this study were not able to compensate entirely for an absence of olfaction with visual information whilst navigating from the Catalonian coast it is perhaps surprising that they were able to locate food successfully without olfaction. It is possible that shearwaters are able to relocate large-scale foraging areas by making use of learned compass directions or local topographic information when departing from the colony. Once reaching these areas, area-restricted search is probably informed by both olfactory and visual information, such as congregations of other seabirds^41^, which could allow anosmic birds to locate resources precisely. Over short time periods, this might be sufficient to exploit resources that are moderately ephemeral in distribution and resolve the apparent contradiction. Over longer time-periods than reported in the current study, the ability to locate large-scale resource areas might decline as resource location has to be found again; a part of the process for which olfaction is likely to be more crucial. Our result is therefore consistent with a complex, multi-sensory search strategy that is likely to begin with a memory-informed outbound departure heading, augmented at different scales by olfaction and vision but the short-term nature of the experiment might preclude a reduction in foraging efficiency of anosmic birds over a longer time scale.

The effect of anosmia on the orientation of shearwaters during homing from distant foraging sites when flying in the absence of local visual cues in this study constitutes further evidence that olfactory information contributes to the map of seabirds navigating over their pelagic environment. These results confirm that in release experiments, differences in anosmic birds’ motivation and foraging ability are unlikely to preclude a real navigational effect of being denied olfactory information and so provide a key progression in our understanding of seabird navigation.

## Electronic supplementary material

Supplementary Information

## References

[1] Gagliardo A (2013). Forty years of olfactory navigation in birds. J. Exp. Biol..

[2] Wallraff HG (2015). An amazing discovery: bird navigation based on olfaction. J. Exp. Biol..

[3] Gagliardo A (2013). Oceanic navigation in Cory’s shearwaters: evidence for a crucial role of olfactory cues for homing after displacement. J. Exp. Biol..

[4] Pollonara E (2015). Olfaction and topography, but not magnetic cues, control navigatin in a pelagic seabird: displacements with shearwaters in the Mediterranean Sea. Sci. Rep..

[5] Mouritsen H, Huyvaert KP, Frost BJ, Anderson DJ (2003). Waved albatrosses can navigate with strong magnets attached to their head. J. Exp. Biol..

[6] Bonadonna F (2005). Orientation in the wandering albatross: interfering with magnetic perception does not affect orientation performance. Proc. Biol. Sci..

[7] Thorup K, Holland RA (2009). The bird GPS - long-range navigation in migrants. J. Exp. Biol..

[8] Wallraff, H. G. *Avian Navigation: Pigeon Homing as a Paradigm*. (Springer-Verlag Berlin Heidelberg, 2005).

[9] Benvenuti S, Gagliardo A (1996). Homing behaviour of pigeons subjected to unilateral zinc sulphate treatment of their olfactory mucosa. J. Exp. Biol..

[10] Budzynski CA, Strasser R, Bingman VP (1998). The effects of zinc sulphate anosmia on homing pigeons, Columba livia, in a homing and a non-homing experiment. Ethology.

[11] Dell’Ariccia G, Bonadonna F (2013). Back home at night or out until morning? Nycthemeral variations in homing of anosmic Cory’s shearwaters in a diurnal colony. J. Exp. Biol..

[12] Schlund W (1992). Intra-nasal zinc sulphate irrigation in pigeons: effects on olfactory capabilities and homing. J. Exp. Biol..

[13] Holland Ra (2009). Testing the role of sensory systems in the migratory heading of a songbird. J. Exp. Biol..

[14] Massa B, Benvenuti S, Ioalè P, Lo Valvo M (1991). Homing of Cory’s shearwaters (Calonectris diomedea) carrying magnets. Boll. Zool.

[15] Bedini C, Fiaschi V, Lanfranchi A (1976). Olfactory nerve reconstitution in homing pigeon after resection - ultrastructural and electrophysiological data. Arch. Ital. Biol..

[16] Bonadonna F, Bretagnolle V (2002). Smelling home: a good solution for burrow-finding in nocturnal petrels?. J. Exp. Biol..

[17] Bonadonna F, Spaggiari J, Weimerskirch H (2001). Could osmotaxis explain the ability of blue petrels to return to their burrows at night?. J. Exp. Biol..

[18] Benvenuti S, Ioalè P (1993). Olfactory experiments on Cory’s shearwater (Calonectris diomedea): The effect of intranasal zinc sulphate treatment on short-range homing behaviour. Boll. Zool.

[19] Guilford, T. C. *et al*. GPS tracking of the foraging movements of Manx Shearwaters Puffinus puffinus breeding on Skomer Island, Wales. *Ibis ***150**, 462–473 (2008).

[20] Tremblay, Y. *et al*. Interpolation of animal tracking data in a fluid environment. *J*. *Exp*. *Biol*. **209** (2005). 10.1242/jeb.01970 16354784

[21] Thiebault A, Tremblay Y (2013). Splitting animal trajectories into fine-scale behaviorally consistent movement units: breaking points relate to external stimuli in a foraging seabird. Behav. Ecol. Sociobiol..

[22] Fayet AL (2015). Lower foraging efficiency in immatures drives spatial segregation with breeding adults in a long-lived pelagic seabird. Anim. Behav..

[23] Biro D, Freeman R, Meade J, Roberts S, Guilford T (2007). Pigeons combine compass and landmark guidance in familiar route navigation. Proc. Natl. Acad. Sci..

[24] Zuur, A., Leno, E., Walker, N., Saveliev, A. & Smith, G. *Mixed Effects Models and Extensions in Ecology with R*. doi:10.1007/978-0-387-87458-6 (Springer-Verlag, 2009).

[25] Satterthwaite FE (1946). An approximate distribution of estimates of variance components. Biometrics Bull..

[26] Bates D, Maechler M, Bolker B, Walker S (2015). Fitting linear mixed-effects models using lme4. J. Stat. Softw..

[27] Boersma PD, Wheelwright NT (1979). Egg neglect in the Procellariiformes: Reproductive adaptations in the fork-tailed storm-petrel. Condor.

[28] Afán I (2014). Foraging movements and habitat niche of two closely related seabirds breeding in sympatry. Mar. Biol..

[29] Neves V, Nolf D, Clarke M (2012). Spatio-temporal variation in the diet of Cory’s shearwater Calonectris diomedea in the Azores archipelago, northeast Atlantic. Deep Sea Res. Part I Oceanogr. Res. Pap..

[30] Ramos JA, Granadeiro JP, Phillips RA, Catry P (2009). Flight Morphology and Foraging Behavior of Male and Female Cory’s Shearwaters. Condor.

[31] Martin AR (1986). Feeding association between dolphins and shearwaters around the Azores Islands. Can. J. Zool..

[32] Cortes V, Manuel Arcos J, Gonzalez-Solis J (2017). Seabirds and demersal longliners in the northwestern Mediterranean: factors driving their interactions and bycatch rates. Mar. Ecol. Prog. Ser..

[33] Dean B (2012). Behavioural mapping of a pelagic seabird: combining multiple sensors and a hidden Markov model reveals the distribution of at-sea behaviour. J. R. Soc. Interface.

[34] Freeman R (2010). Black Petrels (Procellaria parkinsoni) Patrol the Ocean Shelf-Break: GPS Tracking of a Vulnerable Procellariiform Seabird. PLoS One.

[35] Wiltschko R (1996). The function of olfactory input in pigeon orientation: does it provide navigational information or play another role?. J. Exp. Biol..

[36] Guilford T, Taylor GK (2014). The sun compass revisited. Anim. Behav..

[37] Benhamou S, Bonadonna F, Jouventin P (2003). Successful homing of magnet-carrying white-chinned petrels released in the open sea. Anim. Behav..

[38] Wikelski M (2015). True navigation in migrating gulls requires intact olfactory nerves. Sci. Rep..

[39] Nevitt GA, Veit RR, Kareiva P (1995). Dimethyl sulphide as a foraging cue for Antarctic Procellariiform seabirds. Nature.

[40] Dell’Ariccia G (2014). Olfactory foraging in temperate waters: Sensitivity to dimethylsulfide by shearwaters in the Atlantic Ocean and Mediterranean Sea. J. Exp. Biol..

[41] Nevitt G (2000). Olfactory foraging by Antarctic procellariiform seabirds: life at high Reynolds numbers. Biol. Bull..

