# Supplementary material for: Anosmia impairs homing orientation but not foraging behaviour in free-ranging shearwaters
Source: Sci Rep. 2017 Aug 29;7:9668. doi: 10.1038/s41598-017-09738-5 (PMC5575321; doi:10.1038/s41598-017-09738-5)
Supplement: Supplementary file 1 — Supplementary Information [file 41598_2017_9738_MOESM1_ESM.pdf]

Anosmia impairs homing orientation but not foraging behaviour in free-ranging shearwaters

*Padget, O.[a], Dell'Ariccia, G.[b], Gagliardo, A.[c], Gonzalez-Solis, J.[b] & *Guilford, T.[a]

[a] Oxford Navigation Group, Department of Zoology, University of Oxford OX1 3PS

[b] Biodiversity Research Institute (IRBio) & Department of Animal Biology, University of Barcelona, Barcelona, Spain

[c] Department of Biology, University of Pisa, Pisa, Italy

*Correspondence to: oliver.padget@zoo.ox.ac.uk; tim.guilford@zoo.ox.ac.uk

Supplementary Information

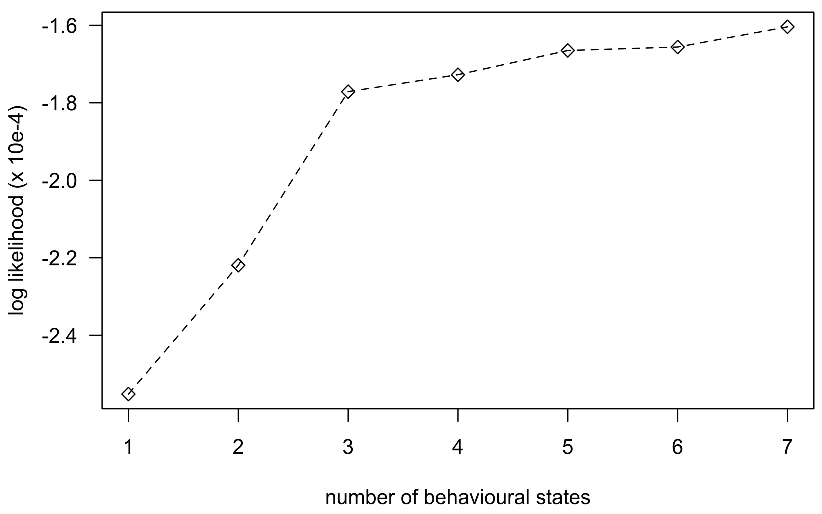

Figure S1. Log-likelihood of Gaussian mixture models with different numbers of putative states. The elbow indicates that a three state mixture model represents the best compromise between explanatory power and model fit. The states in our chosen mixture model were later identified as foraging, resting and commuting.

Table S1. Exploratory statistical analysis between magnetically manipulated birds that retained their magnets (n = 4) until recapture and those that did not (n = 6). The statistical methodology was the same as the main analyses described in the main text.

	**Diurnal activity**	**GLMM**		**Nocturnal activity**	**GLMM**	
	Mean±SE	$X^2(2)$	p	Mean±SE	$X^2(2)$	p
**Foraging**	With magnet: 0.34 ± 0.27<br>Without magnet: 0.35 ± 0.20	0.30	0.59	With magnet: 0.15 ± 0.21<br>Without magnet: 0.22 ± 0.17	2.93	0.12
**Resting**	With magnet: 0.36 ± 0.28<br>Without magnet: 0.36 ± 0.20	0.02	0.89	With magnet: 0.75 ± 0.21<br>Without magnet: 0.64 ± 0.17	1.52	0.22
**Commuting**	With magnet: 0.30 ± 0.26<br>Without magnet: 0.29 ± 0.19	0.19	0.66	With magnet: 0.10 ± 0.17<br>Without magnet: 0.15 ± 0.14	0.70	0.40

	**Pelagic**	**GLMM**		**Coastal**	**GLMM**	
	Mean±SE	$X^2(2)$	p	Mean±SE	$X^2(2)$	p
**Homing orientation**	with magnets: -6.07 ± 6.77<br>without magnets: 5.01 ± 4.00	2.75	0.10	with magnet: -9.74 ± 9.39<br>without magnetic: 3.63 ± 4.87	2.12	0.15

Entire trip

	Mean±SE	t	p
**Mass gained**	with magnets: 116.67 ± 42.06 g<br>without magnets: 61.50 ± 36.44	t(5) = -0.97	0.38

Nearest neighbour method

The nearest neighbour distance (NND) gives a metric, in km, which is the average spatial distance between two tracks and therefore relates to the fidelity of two tracks. NND between two tracks, a focal track and a comparison track, is calculated by taking in turn each GPS fix on the focal track and finding the nearest (in space) GPS location on the comparison track. The distance between these two nearest neighbours is measured; giving each GPS fix on the focal track an NND to its nearest neighbour. The mean NND for the focal track is then taken as the nearest neighbour distance for that track from the comparison track. The distance varies slightly depending on which track is the focal and comparison track and so for the two tracks, the process is done reciprocally and averaged.

Fidelity of homing route to outbound route within each trip among treatments

To test whether treatment had an effect on birds' propensity to retrace their outbound route as they returned to the colony, we computed nearest neighbour distances (as above) between the outbound and homing sections of each trip. The homing section was defined as for other analyses by our Douglas-Peucker algorithm. The outbound route began at the colony. However, when choosing a point to define as the end of the outbound section, we wanted to avoid foraging behaviour close to the onset of homing whilst capturing the route that the shearwater had taken leading up to its position at the start of homing. To do this, we chose the first time that the outbound track came within a threshold distance of the position that the bird started homing (shown in figure S2). For the threshold, we chose 10% of the distance between the colony and the start of homing for each trip since most behaviour outside of this threshold is active flight, rather than foraging or resting. However, since this choice is largely arbitrary, we conducted the analysis at 0% (i.e. all track before the start of homing was compared with all track after the start of homing), 5%, 20% and 50%. We chose to only analyse fixes labelled as in the flying state so that average distances were not heavily weighted by where birds chose to rest. Since the data comprised repeated measures from individual birds, we used linear mixed models to test for an effect of treatment on the NND (fidelity) between the outbound and inbound sections of track. The results are shown in table S3.

Table S2. Table shows nearest neighbour estimates between outbound and inbound routes for each of the three treatments. The analysis is repeated for different thresholds for determining the end of the outbound section.

**Theshold**	**LMM estimates of inbound-outbound NND**	**Likelihood ratio test statistics**	
	Mean ± s.e.	$X^2(2)$	p
**0%**	Control: 32 ± 9 km<br>Magnetic: 21 ± 8 km<br>Anosmic: 19 ± 6 km	2.91	0.23
**5%**	Control: 33 ± 9 km<br>Magnetic: 23 ± 9 km<br>Anosmic: 20 ± 7 km	2.71	0.23
**10%**	Control: 34 ± 10 km<br>Magnetic: 23 ± 9 km<br>Anosmic: 21 ± 7 km	2.59	0.27
**20%**	Control: 34 ± 10 km<br>Magnetic: 33 ± 10 km<br>Anosmic: 21 ± 7 km	2.24	0.33
**50%**	Control: 32 ± 10 km<br>Magnetic: 16 ± 11 km<br>Anosmic: 12 ± 8 km	4.42	0.11

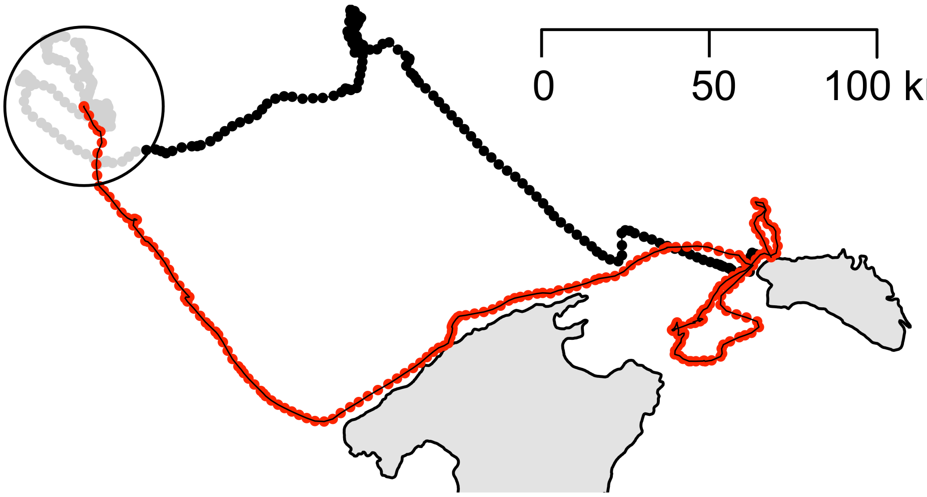

Figure S2. An example track is shown with outbound (black) and homing (red) sections of track. The circle representes the 10% distance contour around the start of homing. The end of the outbound section is defined when the bird first comes within the contour. Maps were generated in R using the "maps" package (Original S code by Richard A. Becker, Allan R. Wilks. R version by Ray Brownrigg. Enhancements by Thomas P Minka and Alex Deckmyn. (2016). maps: Draw Geographical Maps. R package version 3.1.1. http://CRAN.R-project.org/package=maps) and "maptools" package (Roger Bivand and Nicholas Lewin-Koh (2016). maptools: Tools for Reading and Handling Spatial Objects. R package version 0.8-39. http://CRAN.R-project.org/package=maptools).

Repeat trip fidelity within birds

For individual birds that underwent multiple trips during the experiment, we measured the NND pairwise among each bird's trips. For example, a bird which underwent three trips would have the NND for trip 1 and 2, 2 and 3 and 1 and 3 calculated in both directions (the reciprocal) and averaged to give a measure of that bird's repeat trip fidelity. This was done for the outbound sections of trips, from leaving the colony to the furthest point reached from the colony on that trip since this would capture fidelity in foraging destination. This gave within-bird NND measures that could be compared among treatments. Because only 10 birds underwent multiple trips during the experiment, these are exploratory statistics on a small data set (n = 13) but show no evidence of any difference in fidelity across treatments that might have been caused by anosmia.

Table S3. Nearest neighbour distances (NND) are shown for repeat tracks from the same birds, and compared among treatments. There was no difference among treatments (GLM: $F = 0.10$, $df = 2,10$, $p = 0.91$)

	**Mean ± s.e.**
**Control (n=3)**	NND: 37.8 ± 11.4 km
**Magnetic (n = 6)**	NND: 39.6 ± 9.7 km
**Anosmic (n = 4)**	NND: 35.2 ± 7.5 km
